# Tau phosphorylation highlight amyloid precursor protein role in Alzheimer’s disease beyond Aβ production

**DOI:** 10.1002/alz70859_096936

**Published:** 2025-12-25

**Authors:** Beka Solomon

**Affiliations:** ^1^ Tel Aviv university, Tel Aviv Israel

## Abstract

**Background:**

In Alzheimer’s disease (AD) and related neurodegenerative conditions known astauopathies, the tau protein undergoes abnormal hyperphosphorylation and formsintraneuronal neurofibrillary tangles leading to neurodegeneration. Various therapeutic strategies currently focus on specific tau phosphorylation, either by modulating tau kinase activity or through targeted immunotherapy using passive or active vaccination against pathological tau. In this context, we introduced an original approach to reduce phosphorylated tau levels using antibodies against the β‐secretase cleavage site on the amyloid precursor protein (APP). BACE1‐mediated cleavage of APP plays a pathological role in AD beyond Aβ production which shares neurotoxicity with other proteolytic APP fragments as **APP intracellular domain (β‐AICD)** as well extracellular sAPPβ.

**Method:**

Our team developed site‐specific monoclonal antibodies, referred to as ‘blocking β site 1’ (BBS1), which prevent the initiation of endocytic APP processing. We explored the feasibility of intracerebroventricular administration of the monoclonal antibody BBS1 in triple transgenic mice model of AD. After continuous BBS1 administration via mini‐osmotic pumps for one month, cognitive functions were improved and we investigated the effect on **amyloid and tau pathology**

**Result:**

By limiting APP processing via β‐secretase along the endocytic pathway, this method overcomes some of the limitations associated with BACE inhibition . MAb BBS1 does not disaggregate existing plaques but prevents further assembly by depriving Aβ production and mitigating both intraneuronal and extracellular Aβ toxicity. In mice treated with mAb BBS1 compared to the control mAb,was a remarkable **51% reduction in total tau levels**, an impressive **80% decrease in phosphorylated tau** demonstrated through both biochemical and histological analyses using the AT‐8 antibody. The antibody PHF1 revealed a substantial **56% decrease in** paired helical filaments (tangles Additionally, the total levels of GSK3β, a key kinase involved in tau phosphorylation, were significantly reduced by **74%**.

**Conclusion:**

Interactions among GSK‐3β, APP and tau contribute to the cytotoxic effects observed in neuronal cells. These mechanisms may involve transcriptional events within the nucleus, ultimately impacting tau phosphorylation. Understanding these intricate pathways is essential for developing targeted therapies to mitigate tau‐related neurodegeneration.

